# Swift Onset, Swift Recovery: Unusual Nonrheumatic Myocarditis in a Young Adult Post Group A Streptococcal Pharyngitis

**DOI:** 10.1155/2024/4942739

**Published:** 2024-08-12

**Authors:** Andres Rios, Colby Wood, Ricardo Isaiah Garcia, Emily C. Mitchell, Jacob Nichols

**Affiliations:** ^1^ Texas Tech University Health Science Center School of Medicine, Lubbock, Texas, USA; ^2^ University Medical Center Department of Infectious Disease, Lubbock, Texas, USA

## Abstract

This case report highlights the unusual presentation and management of nonrheumatic myocarditis in a 24-year-old male, an age demographic not commonly associated with myocardial complications following Group A streptococcal pharyngitis. The patient, devoid of any prior medical history, manifested symptoms one day after being diagnosed with Group A streptococcal pharyngitis, a stark contrast to the typical progression of myocardial complications. The swift onset of symptoms and the patient's subsequent clinical presentation necessitated a comprehensive diagnostic approach. The patient's symptoms were successfully alleviated with amoxicillin and anti-inflammatory therapy, underscoring its potential efficacy in managing nonrheumatic myocarditis. This case serves as a poignant reminder of the importance of maintaining a broad differential diagnosis, especially in atypical presentations, and the pivotal role of timely clinical intervention. The insights from this report contribute to the broader understanding of nonrheumatic myocarditis, emphasizing the significance of tailored diagnostic and therapeutic strategies to ensure optimal patient outcomes.

## 1. Introduction

Nonrheumatic myocarditis (NRM) is a distinct clinical entity that stands apart from the more commonly recognized acute rheumatic fever (ARF) [1]. Unlike ARF, which typically presents weeks after a Group A streptococcal (GAS) infection, NRM makes its appearance in a much shorter timeframe. This rapid onset of symptoms, coupled with its clinical manifestations, can often lead to diagnostic challenges [2]. ECG changes, particularly ST elevations, further complicate the clinical picture, making it imperative for clinicians to differentiate between an actual myocardial infarction and NRM [2]. The differentiation is crucial, as the management strategies for both conditions differ significantly. While the pathophysiology of ARF is better understood, involving molecular mimicry that triggers cross-reactive immune responses between host cardiac antigens and Streptococcus antigens, the pathophysiology of NRM is less understood and is thought to arise from direct invasion of the myocardium by the GAS organism or through a toxin-mediated mechanism [1, 3]. In contrast to a toxin-mediated pathophysiology, a recent article found that bacteria, neutrophilic infiltrate, and microabscesses were found on histological examination of a patient with recurrent myocarditis on autopsy [4]. Given these diverging conclusions, current literature on the pathophysiology of NRM is poorly understood and therefore further warrants future research to be done [5]. This difference in pathogenesis further underscores the need for a precise diagnosis.

While the immediate treatment might appear similar, the long-term management and follow-up vary [2]. Recent case reports generally indicate that treatment aimed at addressing Streptococcal infection also serves as the primary approach for nonrheumatic myocarditis, often resulting in resolution of symptoms within a few days to one week [6, 7]. This suggests that treatment typically includes antibiotics like penicillin, supplemented occasionally by supportive care such as rest, NSAIDs, and colchicine, which have been endorsed as beneficial management options [6, 8]. Additionally, the rarity of NRM in developed nations often leads to a lower index of suspicion among healthcare providers [2]. However, given the potential severity of the condition and its implications, a heightened awareness and understanding of NRM are essential. This case of a 24-year-old male emphasizes the importance of considering NRM in the differential diagnosis when young adults present with cardiac symptoms following a GAS infection [2].

## 2. Case Presentation

**Figure 1:**
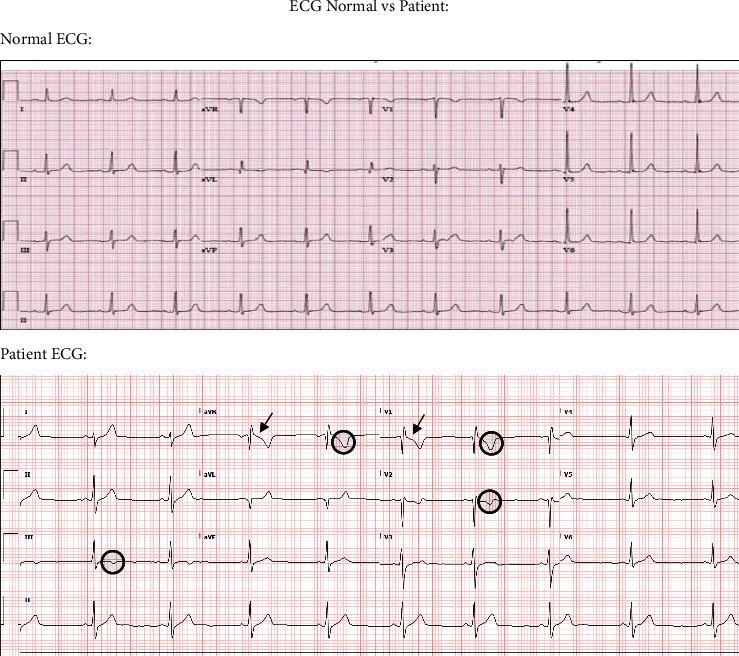
ECG Normal vs. Patient. Patient's ECG demonstrated sinus bradycardia with T wave inversions in leads III, aVR, V1, and V2. Moderate ST depressions were noted in aVR and V1. No progression was noted over time which cardiologist mentioned could be a normal variant of the patient.

The patient is a 24-year-old male with no previous medical history who presented to an urgent care clinic with nonradiating central chest pain onset of a few hours. One day prior, the patient was evaluated at a family medicine clinic for a sore throat where rapid antigen testing was positive for GAS pharyngitis for which he was prescribed amoxicillin 500 mg PO q12 h for 10 days. Vitals at that time were significant only for tachycardia with a heart rate of 116 beats per minute. On arrival to the urgent care, an ECG was performed showing sinus bradycardia with T wave inversions in leads III, aVR, V1, and V2 and ST depressions in leads aVR and V1 can be seen in Figure 1. Subsequently, the patient was escorted to the emergency room (ER) for further evaluation.

In the ER, vital signs again revealed bradycardia, but were otherwise unremarkable. Patient received a one-time dose of aspirin 324 mg PO. Labs were obtained and pertinent results are present in Table 1.

Due to the elevated troponin T value, the patient was started on a heparin drip and cardiology was consulted. Based on their evaluation, it was felt that the patient most likely had myocarditis and less likely acute coronary syndrome. A transthoracic echocardiogram was significant only for trace tricuspid and mitral regurgitation. Additionally, Cardiology reported that ECGs obtained during urgent care and ER could be a normal variant given that repeat ECGs continued to show the same findings. No ECG demonstrated any evidence of acute coronary syndrome.

The infectious diseases team was consulted for evaluation and treatment recommendations as well. Based on their evaluation and timing of chest pain just one day after the diagnosis of streptococcal pharyngitis, it was felt that the patient was likely experiencing nonrheumatic myocarditis rather than acute rheumatic fever, although concurrent viral myocarditis was thought to be a possibility. The patient was initially treated with penicillin G IV 24 MU q24 h for GAS coverage and clindamycin 600 mg PO q12 h to decrease toxin production. Serological studies were ordered for adenovirus, CMV, EBV, echovirus (9, 11, 30), HHV 6 and 8, parvovirus B19, and a respiratory viral panel by PCR. Given clinical improvement over the following two days, the patient was prescribed indomethacin 25 mg TID, colchicine 0.6 mg BID, and amoxicillin 875 mg BID for 5, 7, and 10 days, respectively.

At follow up visits with family medicine and cardiology, symptoms had resolved completely. Echo viral 4, 7, 9, 11, and 30 antibodies were detected in serum during patient's admission into the hospital; however, subsequent convalescent titers were not performed given the patient's symptomatic improvement with antibiotics and anti-inflammatory medications. No long-term pharmacological prophylaxis was indicated at this time due to complete resolution of symptoms. Nonpharmacological treatments included rest and limited physical activity for a few months with slow return to baseline as tolerated. Prognosis included a return to baseline with no future complications. After a year of follow-up appointments, the patient was medically cleared and follow ups were no longer scheduled.

## 3. Discussion

This case brings forth several considerations in the clinical approach and management of NRM, especially in atypical presentations. The patient's experience underscores the importance of considering NRM and its complications in adult populations.

### 3.1. Diagnostic Distinctions between ARF and Nonrheumatic Myocarditis

The diagnostic criteria for ARF and NRM differ significantly, particularly in the context of a recent GAS infection. While ARF typically presents 2 to 3 weeks post-GAS infection, nonrheumatic myocarditis can manifest symptoms in a much shorter timeline [2]. Laboratory findings, such as elevated troponin levels, and specific EKG readings, including ST-segment elevation, are more indicative of nonrheumatic myocarditis [9]. In fact, a study found that cardiac troponin T (cTnT) levels typically remained within normal ranges in patients with acute rheumatic carditis, suggesting that cTnT might not be the most reliable diagnostic marker for ARF [10]. Conversely, other cases discussing the instance of nonrheumatic streptococcal myocarditis presented with progressively rising troponin levels [11, 12]. These distinctions are crucial in the diagnostic setting, as they guide clinicians in differentiating between these two conditions, which, although related to GAS infection, require different therapeutic approaches.

### 3.2. Underrepresentation in Young Individuals

One of the major defining factors in this case is the patient's age. The presentation in a 24-year-old male highlights the necessity for healthcare providers to maintain a high index of suspicion for GAS infection complications across a broader age range. Young individuals presenting with chest pain following a strep infection are often not immediately considered for nonrheumatic myocarditis. The prevailing clinical perception leans towards ARF, especially given its historical association with GAS infections [13]. However, nonrheumatic myocarditis can mimic STEMI presentations in young adults, emphasizing the need for heightened clinical suspicion [2].

### 3.3. The Imperative of Recognizing Nonrheumatic Myocarditis

Despite its rarity, NRM post-GAS infection should be a more prominent differential in patients presenting with the aforementioned symptoms. This case and multiple others emphasize the importance of distinguishing between ARF and NRM, especially given the potential cardiac complications associated with the latter [13–17]. A noteworthy point of mention is that titers were positive for echovirus 4, 7, 9, 11, and 30. These titers were deemed necessary to rule out their role on this patient. Nonetheless, successive titers were not performed due to the pathology resolution when treated with antibiotics and anti-inflammatories. Furthermore, there have been documented instances where nonrheumatic myocarditis poststreptococcal pharyngitis presented as acute ST-elevation myocardial infarction, further emphasizing the clinical significance of this differential [9, 11, 12, 18, 19]. Furthermore, a systematic review of 70 patient studies revealed that approximately half (47.1%) of the patients exhibited ST-segment elevations, with the majority showing other notable EKG abnormalities as well [20]. Interestingly, unlike those cases, which often exhibited ECG patterns resembling acute ST elevated myocardial infarctions, this particular case showed elevated troponin levels alongside a normal ECG reading (due to cardiologist normal variant findings). Therefore, while this case shares similarities with others involving NRM, it stands out due to its clinical presentation with a normal ECG reading.

## 4. Conclusion

In conclusion, while ARF remains a significant concern post-GAS infection, the potential for nonrheumatic myocarditis should not be overlooked, especially in young individuals presenting with chest pain. The diagnostic distinctions between these conditions, coupled with the underrepresentation of nonrheumatic myocarditis in clinical settings, underscore the importance of maintaining a broad differential diagnosis. As the literature suggests, a more inclusive approach can lead to timely and appropriate interventions, ultimately improving patient outcomes.

## Figures and Tables

**Table 1:** Pertinent lab values.

Component	Measured value	Normal Range
WBC	20.66 k/*μ*L (high)	4.5–11.0 k/*μ*L
Erythrocyte sedimentation rate	69 mm/hr (high)	0–15 mm/hr
Brain natriuretic peptide (BNP)	174 pg/mL (high)	<100 pg/mL
Troponin T HS	844.3 ng/L (critical)	<40 ng/L (healthy patients)
C-Reactive protein	23.4 mg/dL (high)	<0.5 mg/dL
Antistreptolysin O (ASO)	55 IU/mL	20–200 IU/mL
DNase-B Ab	<95 units/mL	<85 units/mL
